# The use of clinical scales and PROMs in headache disorders and migraine, summarizing their dissemination and operationalization

**DOI:** 10.1016/j.heliyon.2023.e16187

**Published:** 2023-05-13

**Authors:** Pınar Yalinay Dikmen, Aynur Ozge, Paolo Martelletti

**Affiliations:** a Department of Neurology, Acıbadem University School of Medicine, Istanbul, Turkey; b Department of Neurology, Mersin University Faculty of Medicine, Mersin, Turkey; c Department of Clinical and Molecular Medicine, Sapienza University, Rome, Italy

## Abstract

Measurements are an essential aspect of scientific research. This review will present clinical scales and patient-reported outcome measures (PROMs) for headache disorders and migraine that have been endorsed by the International Headache Society (IHS) and are intended for use by both physicians and researchers. A clinical scale is a tool to assess a patient’s condition or symptoms in a standardized and quantifiable way. Clinical scales are often used in research settings and can be used to track a patient’s progress over time, monitor the effectiveness of treatment, and make decisions. They can be self-administered or completed by a healthcare professional. PROMs are tools used to evaluate a patient’s health status, symptoms, and quality of life. These measures are completed by the patient and provide valuable information about the patient’s perspective and experience of their condition. PROMs are increasingly used in clinical practice and research to improve patient-centered care, patient engagement, and shared decision-making.

This review also briefly covers the creation process, testing for reliability and validity, and interpreting the results of the use of clinical scales and PROMs in clinical and research settings in headache disorders. The first step in creating a clinical scale or PROM is to define the purpose of the scale and the population it is intended to assess. The next step is to identify the domains or areas that the scale will assess. Then, the items or questions that will be included in the scale need to be developed. These items should be relevant to the defined purpose and population of the scale and should be worded clearly and concisely. After the items have been developed, the scale or PROM can be administered to a sample of individuals in the target population. This allows researchers to assess the reliability and validity of the scale or PROM, as well as to make any necessary revisions.

## 1 Introduction

Headache is a very common medical problem and also the most common reason for consultation in primary care as well as neurologic practice [1,2]. The International Classification of Headache Disorders, 3rd Edition (ICHD-3), describes more than 200 headache disorders and contains clear diagnostic criteria for all types of headaches [3]. The ICHD is probably well-known among neurologists; however, probably not all physicians will be familiar with the ICHD.

For the last few decades, awareness of headache disorders has increased due to efforts of international and national headache societies and non-profit organizations. Despite this effort, underdiagnosis, and undertreatment of headache disorders, especially migraine, have been well-known for a while. Global Burden of Disease (GBD) studies annually show that headache disorders are very prevalent but highly disabling conditions worldwide [4]. In GBD 2019, migraine was the first cause of disability among women in the 15–49 years age group [5]. As is known, headache disorders can affect various aspects of a patient’s life, including occupational, academic, social, leisure, and family lives and responsibilities. They can cause both increased ictal and inter-ictal burden as well as economic loss because of the costs of medical treatment and lost productivity.

Thanks to many efforts of national headache societies and GBD, headache disorders and migraine are issues of increasing attention and recognition around the world. A PubMed search of “headache” revealed 49,605 results for the last 10 years, among which 19,001 results were related to “migraine”. Clinical trials are a major source to collect evidence and create an evidence-based treatment for diseases. The International Headache Society (IHS) has updated guidelines for controlled trials of acute and preventive treatment of headache and migraine for nearly 30 years. Evaluation of both primary and secondary endpoints has a critical role in the quality of headache studies. Table 1 shows the recommended primary and secondary as well as pharmacoeconomic endpoints of the IHS for controlled trials of migraine treatment in adults [[6], [7], [8]]. The IHS suggests some clinical scales for measuring pain intensity in primary endpoints. Secondary outcomes include researching depression and anxiety with validated scales and using patient-reported outcome measures (PROMs) for the assessment of functional impairment, health-related quality of life (HRQoL), headache impact, and headache-related disability. To evaluate pharmacologic endpoints in headache studies, the Work Productivity and Activity Impairment (WPAI) instrument, which measures the direct and indirect costs of headache and migraine, is recommended by the IHS.

**Table 1:** Recommended clinical scales and PROMs to capture endpoints of guidelines of the International Headache Society for controlled trials of acute and preventive treatment of migraine in adults.

Guidelines of IHS	Acute treatment	Preventive treatment of EM	Preventive treatment of CM
**Primary endpoints**			
Headache intensity	4-point categorical scale 11-point NRS VAS		
**Secondary endpoints**			
Headache intensity		4-level categorical scale 11-point NRS	11-point VRS 4-level categorical scale
Peak headache pain intensity		11-NRS 4-level categorical scale	11-point VRS 4-level categorical scale
Global evaluation	PGIC		
Global impact (Functional disability and quality of life)	FIS MPFID 24-h MSQoL Minor Symptoms Evaluation Profile		
Associated symptoms (Nausea and vomiting, photophobia, phonophobia)	Verbal Scales of severity		
Validated scales for depression		PHQ-9, BDI, HADS	PHQ-9, PHQ-4, BDI, HADS
Validated scales for anxiety		HADS, STA-I, GAD-7	HADS, STA-I, GAD-7
Scales for suicidal ideation		C-SSRS	
PROMs		PGIC, FIS, MFIQ, MPFID	PGIC, FIS, MFIQ
Healthcare outcomes		MSQ v.2.1., HIT-6, MIDAS, EQ-5D, SF-36	MSQ v.2.1., HIT-6, MIDAS EQ-5D, SF-36
**Pharmacological endpoints**		WPAI instrument	WPAI instrument

IHS: International Headache Society, PROMs: Patient-reported outcome measures, EM: Episodic migraine, CM: Chronic migraine, NRS: numerical rating scale, VAS: Visual Analog Scale, VRS: Visual Rating Scale, PGIC: The Patient Global Impression Scale, FIS: The Functional Impairment Scale, MPFID: The Migraine Physical Function Impact Diary, MSQoL: Migraine specific quality of life, PHQ-4: Patient Health Questionnaire-4, PHQ-9: The Patient Health Questionnaire-9, BDI: Beck Depression Inventory, HADS: The Hospital Anxiety and Depression Scale, STA-I: The State-Trait Anxiety Inventory, GAD-7: The Generalized Anxiety Disorder, C-SSRS: Columbia-Suicide Severity Rating Scale, MFIQ: Migraine Functional Impact Questionnaire, MSQ v.2.1.: The Migraine-Specific Quality of Life questionnaire, HIT-6: The Headache Impact Test, MIDAS: Migraine Disability Assessment questionnaire, EQ: 5D: The EuroQoL-5, SF-36: The Short Form 36-Item Health Survey, WPAI: The Work Productivity and Activity Impairment.

PROMs, as defined by the United States Food and Drug Administration (FDA), are assessments of a patient’s health status that are collected directly from patients, without interpretation by a physician or anyone else; the patient’s response should be considered as recorded [9]. PROMs are also endorsed in the reporting of health measurement instruments (COSMIN statement), diagnostic accuracy studies (STARD statement), systematic reviews (PRISMA statement), observational studies in epidemiology (STROBE statement), and studies conducted using routinely collected observational health data (RECORD statement) [[10], [11], [12], [13], [14], [15]].

This review will focus on promoting clinical scales and PROMs related to headache and migraine that are recommended by the IHS guidelines for both physicians and researchers. Information about the creation, reliability, and validity testing, interpretation, and advantages of using these clinical scales and PROMs in clinical practice and research settings will also be discussed.

## 2 Dissemination and operationalization of clinical scales and PROMs

Measurements play a crucial role in scientific research across various fields, including natural, social, and health sciences [16]. There are many clinical conditions, psychological behaviors, attitudes, and beliefs that are challenging to quantify and measure accurately in the field of biomedical sciences. These may include the severity of a disease, HRQoL, sense of pain, anxiety, and others. These are difficult to measure because there is no “mechanical” scale that can be used to do so. To try to measure and quantify certain qualities, researchers create tools called “scales” that are made up of different elements. Clinical scales are tools in the medical field used to evaluate various aspects of a patient’s health. These scales can be used to measure a wide range of variables, including physical and mental health symptoms, functional ability, and overall quality of life. Different from PROMs, clinical scales are usually administered by trained healthcare professionals, such as physicians or nurses, and are usually used as part of an assessment process to help inform treatment decisions.

Translating a subjective experience such as headache into an objective context is complex and challenging. The task of creating a series of indicators that can lead different individuals to a common understanding of the objective context of headache requires even more advanced levels of expertise and skill. At this stage, the use of clinical scales and PROMs helps physicians as well as researchers. It is crucial for the accuracy of diagnosis, patient referral, and treatment processes that any tool developed for measuring subjective variables in clinical settings needs to be capable of making accurate decisions. To make accurate decisions, the necessary tools must include a valid measurement procedure and assessment process using criteria that are appropriate for the task at hand. Established measurement tools with reliability and validity are essential for the development of valid measurement procedures. Reliability refers to the consistency of the measurement and validity refers to the ability of the measurement to accurately assess the intended variable, without contamination from other factors [17,18].

**Fig. 1:**
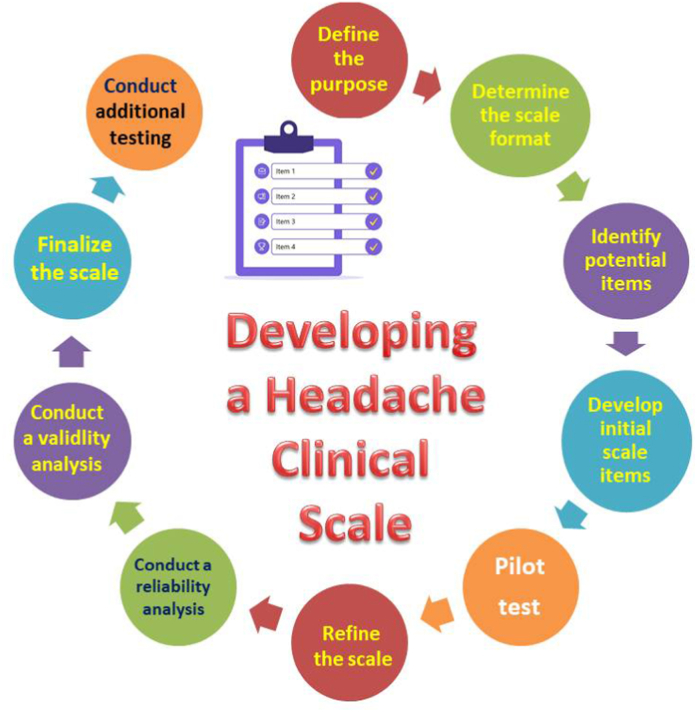
Recommended steps for development of a clinical scale for headache disorders. A Step-by-Step Model for Developing a Clinical Scale for Headache Disorders: 1. Define the Purpose of the Scale: The first step is to clearly define the purpose of the clinical scale. What type of headache disorder is the scale intended to measure? Is it intended for clinical diagnosis, severity assessment, or treatment monitoring? 2. Determine the Scale Format: The next step is to determine the format of the scale. Will it be a self-report scale, an observer-rated scale, or a combination of both? Will it be a Likert scale, a visual analog scale, or another type of scale? 3. Identify Potential Items: Based on the purpose of the scale, identify potential items that could be included. This may involve reviewing the literature on headache disorders or consulting with experts in the field. 4. Develop Initial Scale Items: Based on the identified items, develop the initial set of scale items. It is important to ensure that the items are clear and unambiguous. 5. Pilot Test the Scale: Pilot test the scale on a small sample of patients with headache disorders. This will help identify any issues with the scale, such as unclear items or response options. 6. Refine the Scale: Based on the results of the pilot test, refine the scale items and response options. This may involve removing or modifying items or adding new items. 7. Conduct a Reliability Analysis: Test the reliability of the scale by analyzing the consistency of responses to the scale items. This may involve calculating Cronbach's alpha or other measures of internal consistency. 8. Conduct a Validity Analysis: Test the validity of the scale by examining its ability to measure what it is intended to measure. This may involve comparing the scale scores to other measures of headache disorder severity or diagnosis. 9. Finalize the Scale: Based on the results of the reliability and validity analyses, finalize the scale. This may involve making further refinements or adjustments. 10. Conduct Additional Testing: Conduct further testing of the scale on larger and more diverse samples of patients with headache disorders to ensure that the scale is applicable to a wider range of individuals.

The process of creating clinical scales and PROMs typically involves several steps (Fig. 1). In clinical practice, a three-step approach is recommended for the development and validation of a scale: first, establish the conceptual framework based on the main concept; second, construct the item pool through reduction and scale development; and finally, perform field testing to determine the scale’s validity and reliability. Creating a scale idea based on a conceptual framework should begin with an in-depth literature review. Then, the authors need to construct semi-structured patient interviews and consult with an expert panel to determine the optimal items. After that, the scale should be administered to appropriate patient groups and controls for further evaluation. In this step, it is necessary to consider inter-research variability and test-retest reliability and to administer the clinical scale or PROM accordingly. Afterward, appropriate statistical methods should be applied to analyze the data for validity and reliability [19,20]. Most PROMs commonly used in migraine treatment did not follow the FDA’s recommended development process, particularly the steps involving patient participation.

The minimal clinically important change (MCIC) and the minimal clinically important difference (MCID) are both measures used to determine the smallest change in a clinical outcome that is meaningful to patients or healthcare providers. This information is crucial for physicians to be able to interpret changes in an individual patient’s score on the instrument [[21], [22], [23]]. A glossary of relevant terminology is provided here: https://www.fda.gov/drugs/development-approval-process-drugs/patient-focused-drug-development-glossary. The difference between two measures lies in the type of outcome measure being used. The MCIC is used for continuous outcome measures, such as pain scores, whereas the MCID is used for clinical scales, such as quality of life or functional status. For example, in a study measuring the effectiveness of pain medication, the MCIC may be the smallest change in pain score that patients perceive as a meaningful improvement in their pain. The MCID is important in interpreting change scores on PROMs such as HRQoL questionnaires because it helps to determine whether a change in the score on the questionnaire represents a clinically meaningful change in the patient’s condition. The MCID can be determined through various methods, including anchor-based methods (e.g. using a global rating of change), distribution-based methods (e.g. using the standard deviation of the change scores), and patient-based methods (e.g. using patient’s self-rated change). Calculating the MCID helps to ensure that a change score on a measure is interpreted in the context of what is meaningful to the patient, rather than just a change in score that may not be meaningful. This can be especially useful in conditions such as headaches, where the impact of the condition on the patient’s daily life may be more important than the actual severity of the headache itself [24,25].

## 3 The use of clinical scales and PROMs in headache disorders and migraine

**Fig. 2:**
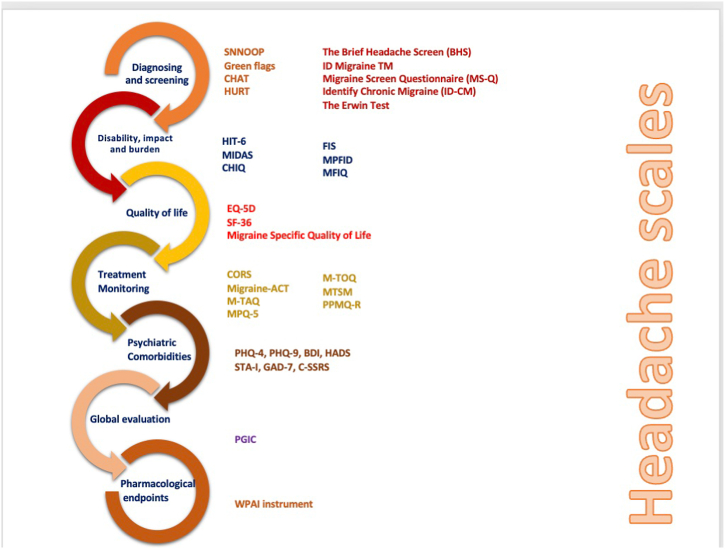
Many aspects of headache disorders can be evaluated clinical scales and PROMs.

Clinical scales and PROMs can be used to exclude secondary headache disorders, screen for primary headache disorders, evaluate HRQoL, assess headache-related disability, detect co-morbidities, and monitor and optimize treatment in headache disorders or migraine (Fig. 2). These instruments can be useful in research studies to assess the effectiveness of different treatment approaches and identify potential risk factors for developing chronic headaches. With these instruments, researchers can quantify and compare the severity of headaches between different groups of people and track changes in headache severity over time. This information can help to identify patterns and trends that may be useful in developing more effective treatments for headache disorders.

### 3.1 Diagnosing and screening clinical scales and PROMs in headache disorders and migraine

There are several instruments can be used to help identify and diagnose headache disorders and migraine (Table 2). These tools can be helpful in situations where there is insufficient time to fully evaluate a person’s symptoms, such as in an emergency department (ED) or primary care setting. In research studies, these tools can be used to quickly identify and include a large number of participants.

**Table 2:** Screening tools for headache disorders and migraine.

Diagnostic tools	Purpose of development	Format
**SNNOOP**	Secondary headaches Covers 15 red flags in headache	Interview with a trained health care professional
**Green flags**	Primary headaches Covers 5 green flags in headache	Interview with a trained health care professional
**The Computerized Headache Assessment Tool (CHAT)**	Primary headaches	Internet-based program with branching logic
**The Headache Under-Response to Treatment (HURT)**	Headache frequency, Headache-related disability, Acute medication use, Perceived control of headaches, Knowledge and the understanding of diagnosis	8-item questionnaire
**Screening tools**		
**The Brief Headache Screen (BHS)**	Migraine and CDH and MOH	4-item questionnaire
**ID Migraine**^**TM**^	Migraine	3-item questionnaire
**Migraine Screen Questionnaire (MS-Q)**	Migraine	5-item questionnaire
**Identify Chronic Migraine (ID-CM)**	Chronic migraine	12-item questionnaire
**The Erwin Test**	Cluster headache	3-item questionnaire

CDH: Chronic daily headache, MOH: Medication overuse headache.

The IHS has a special group that has created lists of red flags, which indicate a secondary etiology, and green flags, which indicate a primary etiology [[16], [17], [18], [19], [20], [21], [22], [23], [24], [25], [26], [27], [28]]. Indeed, most people who experience headaches have a primary headache disorder. In these cases, further diagnostic examinations are usually unnecessary and can be a waste of resources. Only a small percentage (less than 6%) of neuroimaging studies performed on people who come to the ED with a headache as their main symptom show any evidence of a secondary cause [29]. Despite this, it is important to always rule out the possibility of a serious underlying condition.

The SNNOOP10 list is a tool developed by the IHS to help healthcare providers identify red flags that may indicate a secondary headache disorder, rather than a primary headache disorder [26]. If a patient exhibits one or more red flags, it may be necessary to perform a further evaluation to determine if a secondary headache disorder is present. Green flags are symptoms that suggest a patient is more likely to have a primary headache disorder rather than a secondary one [27]. Red flags and green flags should be evaluated by a trained healthcare professional.

Five screening tools have been created for headache and migraine, the Brief Headache Screen (BHS), ID Migraine™, the Migraine Screen Questionnaire (MS-Q), Identify Chronic Migraine (ID-CM), and the Erwin Test. The BHS is a self-administered tool that patients can use to screen their own migraine and medication overuse headache (MOH) and to evaluate their satisfaction with pharmacologic treatment [30]. ID Migraine is a widely used, popular screening tool for migraine [31]. The 3-item questionnaire asks about nausea, photophobia, and disability. The ID Migraine, with two out of three questions being positive, demonstrated a sensitivity of 0.81, a specificity of 0.75, and a positive predictive value of 0.93. There was a high level of agreement between the ID Migraine and the BHS, with 82.6% agreement in the identification of migraine (95% CI: 77.8–87.4%) [32]. A systematic review and meta-analysis of 13 studies involving 5866 patients showed that the pooled estimate for sensitivity (95% CI: 0.75–0.90) and specificity was (95% CI: 0.69–0.83) [33]. The Erwin Test is a brief screening tool. It was created for cluster headache [34]. It is a 3-item questionnaire that assesses pain intensity, duration, and accompanying autonomic symptoms.

The MS-Q is a self-report assessment tool used to identify migraine. It consists of five questions regarding headache frequency, duration, nausea, sensitivity to light or noise, and disability [35]. Láinez et al. reported that the question about sensitivity to light was the most specific, but also the least sensitive, and the question about disability had the highest sensitivity. A separate study of 9670 patients from 410 primary care centers in Spain showed that the sensitivity was 0.82 and specificity was 0.97 in identifying undiagnosed migraine [36].

Finally, the ID-CM is used to evaluate the frequency, accompanying symptoms, medication use, disability, and impact on daily activities regarding migraine [37]. A validation study found that it had a sensitivity of 0.81 and a specificity of 0.89 for detecting migraine. A shorter, 6-item version of the ID-CM was also tested and found to have high specificity (0.93) and moderate sensitivity (0.71) for identifying CM in a real-world setting [38]. However, the 12-item version was ultimately preferred because it had a higher sensitivity and provided additional information on MOH and the impact of headache.

### 3.2 Disability, impact, and burden of headache disorders and migraine

Headache affects many people worldwide, an estimated 4.6% (3.9–5.5%) of adults have frequent headaches, defined as occurring on 15 or more days per month [39]. Headache disorders cause significant disability and negatively impact the quality of life, and productivity, they can also lead to financial strain for both individuals and society [40].

Several instruments have been created to measure the disability, impact, and burden of headache disorders and migraine (Table 3 and Table 4). The Headache Impact Test-6 (HIT-6) is a frequently used PROM, and it is recommended by the IHS as one of the secondary endpoints for controlled trials of both episodic migraine (EM) and CM. HIT-6 was created to measure the impact of headache on a person’s ability to function at work, school, and in social situations [41]. An international team of headache experts from neurology and primary care medicine, in collaboration with the psychometricians who created the Short Form-36 (SF-36) health assessment tool, developed HIT-6. The questionnaire, consisting of six items, and possessing strong psychometric validation against a substantial item pool, can accurately distinguish between migraine and non-migraine headaches. Items in HIT-6 cover several health outcomes from pain to emotional distress. The recall period for HIT-6 is 4 weeks. HIT-6 is easy to use in clinical practice and clinical trials or observational studies for screening and monitoring treatment in patients with headache, including migraine. Studies have shown that the between-group MCID for HIT-6 is a decrease of at least 2.3 points for CM and 1.5 points for EM (24,42). The decrease in scores of the within-patient MCID has been reported as ≥5.0 points [42]. The within-person MCIC has been estimated to be between 2.5 and 6 points. HIT-6 has been translated and validated in many languages.

**Table 3:** Clinical scales and PROMs regarding disability, impact, and burden of headache disorders.

Diagnostic tools	Purpose of development	Format
**Headache Activities of Daily Living Index (HADLI)**	To measure the disability regarding headache focusing exclusively substantial daily life activities over the preceding 4 weeks	9-item questionnaire
**Headache-Attributed Lost Time (HALT-7-30**–**90) Index**	To assess headache-related lost productive time for all headache disorders as well as migraine Three forms of the HALT evaluate the past 7 days, 1 month and 3 months	5-item questionnaire
**Headache-Attributed Restriction, Disability, Social Handicap, and Impaired Participation (HARDSHIP)**	Multi-modular design	Interview with a trained health care professional
**Headache Disability Questionnaire (HDQ)**	To measure headache-specific disability over the past 3 months It is designed for patients who are under physiotherapy for their headaches	9-item questionnaire
**Henry Ford Headache Disability Inventory (HDI)**	To assess measuring both ictal and interictal burden of headache. The Spouse HDI is similar to the HDI, "I" was changed to "my spouse".	25-item questionnaire
**Headache Impact Test (HIT-6)**	To measure an impact of headache on functional health and well-being of headache patients over the preceding 4 weeks	6-item questionnaire
**Headache Impact Questionnaire (HImQ**)	To evaluate the pain intensity and limitations of activity because of headache over the preceding 3 months	16-item questionnaire
**Headache Needs Assessment Survey (HANA)**	To assess the frequency and the most bothersome symptom due to migraine as well as other types of headaches over the preceding 4 weeks	7-item questionnaire
**The Headache Under-Response to Treatment (HURT)**	To create an assessment of headache and decision-making for better headache management	8-item questionnaire

**Table 4:** Clinical scales and PROMs regarding disability, impact, and burden of migraine.

Diagnostic tools	Purpose of development	Format
**Functional Assessment in Migraine (FAIM) Questionnaire**	To measure the impact of migraine on functional status and the level of impairment during daily activities	9-item questionnaire
**Migraine Functional Impact Questionnaire (MFIQ)**	To assess the impact of migraine on physical, social, and emotional functioning in the previous 7 days	26-item questionnaire
**Migraine Disability Assessment Questionnaire** (**MIDAS**)	To determine the grade of disability caused by migraine in adults over the past 3 months Additional two questions (A and B) have inquired about headache frequency and pain severity	5-item questionnaire
**The Pediatric Migraine Disability Assessment Score (PedMIDAS)**	To determine the grade of disability caused by migraine in children and adolescents	5-item questionnaire
**Migraine Interictal Burden Scale-4 (MIBS-4)**	To measure interictal migraine-related burden of the four following areas: impairments at work or school, disruption in family and social life, difficulty in making plans or commitments, and emotional/affective and cognitive distress over the preceding 4 weeks	4-item questionnaire
**Migraine Severity (MIGSEV) Scale**	To assess the migraine severity	7-item questionnaire
**Migraine Physical Function Impact Diary (MPFID)**	To measure the migraine impacts on physical functioning and social interactions over the past 24 h	13-item questionnaire

The Migraine Disability Assessment Test (MIDAS) is widely used in both clinical practice and academic papers. It was created to measure and follow up on migraine-related disability [43,44]. MIDAS is one of the recommended instruments by the IHS for use as an outcome measure for adult migraine preventive studies. The recall period for MIDAS is 3 months. MIDAS was created to measure just the ictal burden of migraine but not in between attacks. The score is a combination of the responses to five questions, with low scores indicating minimal-to-no disability and high scores indicating moderate-to-severe disability. For evaluating migraine-related disability, the first and second questions of MIDAS are intended to evaluate paid or school/work, the next two questions for household chores, and the last question for leisure time. An additional two questions of MIDAS (A and B) ask about headache frequency (number of days with headache in the previous 3 months) and pain severity (average pain severity of headache attacks) in a 0 (no pain at all) to 10 (pain as bad as it can be) scale. For MIDAS, there is no established MCID [45]. The MCIC for MIDAS after non-pharmacologic treatment has been determined as 4.5 points [46]. A pediatric version of MIDAS has been developed [47] and validated for ages 4–18 years. PedMIDAS is like the adult version; however, a major drawback of the scale is that it relies on the child’s recall over the past 3 months.

The Functional Impairment Scale (FIS) is a 4-point scale that assesses the functional status and the level of impairment during daily activities [48,49]. Patients should record their functional disability status before taking any medication. Functional impairment during the performance of daily activities is graded using FIS as 0- no disability, 1- mild impairment, 2- moderate impairment, and 3- severe impairment.

The Migraine Physical Function Impact Diary (MPFID) was created to measure how migraine impacts a patient’s physical functioning including movement, daily tasks, and social interactions in the past 24 h [50,51]. It was developed in accordance with guidance from the FDA for including PROMs in clinical trials to support labeling claims [9]. The creation and validation of the MPFID involved input from experts in PROMs development and experts in treating patients with migraine. The MPFID has 13 questions that include two sections: “Impact on Everyday Activities” with seven questions, and “Physical Impairment” with five questions. Additionally, there is a single question that asks for an overall evaluation of the impact of migraine on daily activities. This tool has demonstrated strong performance in terms of its psychometric properties [52]. The MPFID has been shown to be effective in measuring the impact of interventions for migraine in clinical trials and real-world settings. This PROM allows the assessment of the effect of migraine on both ictal days and interictal days. Additionally, the MPFID has been recognized as valid by the FDA in the United States of America (USA) and is included in the prescribing information for migraine treatments.

The Migraine Functional Impact Questionnaire (MFIQ) was created to evaluate the impact of migraine on physical, social, and emotional functioning in the previous 7 days [53,54]. The MFIQ PROM is promoted by the IHS as one of the secondary endpoints for controlled trials of migraine treatment in adults. It was designed as a regulatory guideline of the FDA to capture treatment effects from a patient perspective in research and clinical practice settings. The MFIQ (version 2) consists of 26 items and has four domains for yielding the impact of migraine: Physical Function (PF), Usual Activities (UA), Social Function (SF), and Emotional Function (EF), and a global item named Overall Impact on UA. The recall period of the MFIQ is the past 7 days. It is important to capture the week-to-week variability as well as to reduce the potential recall bias. The MFIQ has been translated and validated into 20 languages to date.

### 3.3 Quality of life in headache disorders and migraine

HRQoL is a specific aspect of an individual’s overall quality of life that includes their physical and mental health, functional abilities, and overall well-being [55]. HRQoL has been evaluated in patients with headache using two types of questionnaires: generic and migraine-specific. In the IHS guidelines for migraine preventive treatments, it is recommended to use the following three questionnaires: EuroQoL Quality of Life Scale (EQ-5D), SF-36, and Migraine Specific Quality of Life (MSQ v2.1) version 2.1. Migraineurs have lower HRQoL compared with individuals without chronic diseases [56,57]. Studies from the USA and the Netherlands found that migraineurs had a significantly lower HRQoL compared with a control group as measured using the SF-12 and SF-36 questionnaires [58,59]. The ED-5D is a commonly used tool for assessing an individual’s HRQoL [60]. It is composed of two sections: a descriptive system and a visual analog scale (VAS). A study using the ED-5D, the WPIQ, and the Hospital Anxiety and Depression scale showed that both severe migraine and severe asthma could lead to a decline in quality of life, work productivity, and mental well-being [61]. However, the specific areas affected by the diseases differ, with severe migraine having a greater effect on mental health and severe asthma having a greater impact on physical health. In the headache population, reliability, and validity regarding the EQ-5D are still limited [62].

The SF-36 was created to evaluate various symptoms regarding common diseases [63]. In the headache population, the SF-36 has acceptable evidence of construct validity [17]. A population-based case-control study compared patients with and without migraine. The results showed that eight of the nine HRQoL domains of the SF-36 were impaired in migraineurs [64]. The MSQ v2.1. is a disease-specific PROM instrument that measures the impact of migraine attacks on daily activities, divided into three main areas as follows: role-function-restrictive (RR, seven items), role-function-preventive (PR, four items), and emotional functions (EF, three items) with a 4-week recall period [65,66]. The MCID between groups for the MSQ domains of the MSQ-RR, MSQ-PR, and MSQ-EF are 3.2, 4.6, and 7.5 points, respectively [67]. The MSQ v2.1 PROM was frequently used in recent research on monoclonal antibodies [68].

### 3.4 Treatment monitoring in headache disorders and migraine

Table 5 shows the instruments that can be used for monitoring headache disorders. The instruments regarding treatment monitoring help physicians with both initial treatments and follow-up of patients. None of them has been suggested by the IHS guidelines for controlled studies so far. The Migraine Prevention Questionnaire (MPQ-5) facilitates the practical application of the United States Headache Consensus Guidelines for using preventive pharmacotherapy for managing migraine in clinical practice [69].

**Table 5:** Treatment monitoring in headache disorders and migraine.

Diagnostic tools	Purpose of development	Format
**Completeness of Response to migraine therapy (CORS)**	To assess effectiveness of migraine treatments	It is a composite tool. It has 2 domains: static and comparative
**Migraine Assessment of Current Therapy (Migraine-ACT)**	To evaluate the effectiveness of current migraine treatment	13-item questionnaire
**Migraine-Treatment Assessment Questionnaire (M-TAQ)**	To evaluate the effectiveness of current migraine treatment	9-item questionnaire
**Migraine-Treatment Optimization Questionnaire (M-TOQ-15)**	To evaluate the effectiveness of current migraine treatment in research setting	15-item questionnaire
**Migraine-Treatment Optimization Questionnaire (M-TOQ-5)**	To evaluate the effectiveness of current migraine treatment in clinical setting	5-item questionnaire
**Migraine-Treatment Satisfaction Measure (MTSM)**	To assess a patient’s satisfaction with the current migraine treatment	10-item questionnaire
**Patient Perception of Migraine Questionnaire-Revised (PPMQ-R)**	To evaluate a patient’s satisfaction with acute migraine treatment	32-item questionnaire

The Migraine Treatment Satisfaction Measure (MTSM) and Migraine Questionnaire-Revised (PPMQ-R) have good evidence of construct validity from quality studies [18,70]. The MTSM is a reliable and accurate tool to measure patient satisfaction with various migraine treatment options. It is important to note that although the MTSM can provide valuable information about patient satisfaction, it is not a measure of treatment effectiveness or efficacy. The PPMQ-R evaluates the level of patient satisfaction with treatment for acute migraine attacks in five areas: adverse effect discomfort, ease of use, cost, functionality, and efficacy. The Migraine Prevention Questionnaire (MPQ-5) evaluates the frequency of migraine, the use of quick relief measures, the impact of migraine on various aspects of life, and concerns and anxiety associated migraine. Scores are added up to determine a final score, which then falls into one of three categories: no need for preventive treatment, consider preventive treatment, or suggestive treatment. In addition, each question has its own threshold score, which may trigger a warning or alert a physician to perform further evaluations in this specific area. Completeness of Response to migraine therapy (CORS) is a PROM, and it can be used in both clinical practice and research settings to assess the effectiveness of migraine treatment [71]. CORS can show that new migraine treatments are more effective than older ones. It is important to note that CORS can provide valuable information on treatment effectiveness, but it may not capture all aspects of a patient’s satisfaction with their treatment, which is why it is often used in conjunction with PROMs such as the MTSM to obtain a more comprehensive evaluation of the treatment.

### 3.5 Detecting comorbidities in headache disorders and migraine

Migraine is a chronic neurologic disorder that is often accompanied by comorbidities, including cardiovascular disorders (i.e., stroke, myocardial infarction), psychiatric disorders (i.e., depression, anxiety, panic disorder, bipolar disorder, personality disorders, suicide attempts), neurologic diseases (i.e., epilepsy), sleep conditions (i.e., insomnia, restless leg syndrome, sleep apnea, poor sleep quality and duration), inflammatory conditions (i.e., allergic rhinitis, asthma) and chronic pain conditions (i.e., fibromyalgia) [[72], [73], [74], [75], [76]]. These comorbidities can have a significant impact on the patient’s quality of life and can also affect the course and treatment of migraine [77]. Therefore, it is important to identify and treat these comorbidities to improve the patient’s overall health and well-being and improve the effectiveness of treatment. Moreover, comorbidities have been linked to an increased risk of migraine becoming chronic and multiple comorbidities are associated with an increased risk of MOH and the development of new cases of chronic migraine [[78], [79], [80]].

The guidelines of the IHS for controlled trials of preventive treatment of migraine attacks in EM and CM suggest using validated scales for depression and anxiety (Table 1). In this review, we focus only on scales regarding psychiatric comorbidities. The Patient Health Questionnaire-4 (PHQ-4) is a brief screening tool used to identify individuals who may be experiencing symptoms of depression or anxiety and as a brief follow-up assessment for individuals who have already been diagnosed as having these conditions [81,82]. The reliability and validity of the PHQ-4 in the general population are good. The questions on the PHQ-4 assess the frequency of symptoms of depression and anxiety over the past two weeks. PHQ-9 is reliable and valid for screening and severity evaluation in diagnosed patients and it can be used in both primary and specialist settings [83,84]. A validation study of the PHQ-9 for detecting major depressive disorder in patients with migraine has been performed [85]. The results of the study showed that the cut-off score of the PHQ-9 was different from reported values in published studies of primary care patients.

The Beck Depression Inventory-II (BDI) is a widely used tool that assesses the severity of depression in patients by asking them to indicate their level of 21 different symptoms over the past 2 weeks [86]. This version of the scale has been found to have strong content and construct validity and a positive correlation with other validated tools such as the Hamilton Psychiatric Rating Scale (HRSD) and the Minnesota Multiphasic Personality Inventory (MMPI) among migraineurs.

The Hospital Anxiety and Depression Scale (HADS) has two subscales, one for anxiety (HADS-A) and one for depression (HADS-D), each consisting of seven items [87]. HADS has been found to have high-level reliability and validity. It is particularly useful as a tool for screening for anxiety and depression in non-psychiatric populations such as medical patients, including patients with headache or migraine.

The State-Trait Anxiety Inventory (STAI) is a psychological tool used to measure an individual’s level of anxiety, reflecting how they are feeling at the time they take the test [88]. The STAI consists of two different 20-item self-report scales: the State Anxiety Scale (STAI-S) and the Trait Anxiety Scale (STAI-T). The STAI should be administered and interpreted by a trained professional because it can be affected by various factors, and the results are not definite and must be complemented with other evaluations.

Generalized Anxiety Disorder-7 (GAD-7) was created as a screening instrument for capturing GAD in primary care settings [89]. The reliability and validity of both the GAD-7 and GAD-2 have been shown in the migraine population [90].

The Columbia-Suicide Severity Rating Scale (C-SSRS) is a standardized tool used to assess the risk of suicide and self-harm in individuals [91]. It is a self-report measure that can be used in a variety of settings, including clinical practice, research, and schools. Its results are used to guide treatment decisions and interventions. The C-SSRS has been used in headache studies [92,93].

### 3.6 Global evaluation

The Patient Global Impression of Change (PGIC) scale is a useful instrument in the clinic for measuring patients’ overall subjective perception of their illness, including the severity of symptoms, functional impairment, and overall well-being [94]. It is a single-item measure that asks patients to rate their overall impression of their illness on a 7-point scale. The PGIG is frequently used in clinical studies regarding monoclonal antibodies in migraine to evaluate the effectiveness of interventions. To account for the high placebo effect frequently seen in studies of migraine, the PGIC scale uses the two highest categories of improvement, “very much improved” and “much improved,” as the benchmark for determining improvement in the treatment group.

### 3.7 Pharmacologic endpoints

The WPAI scale is used to measure the impact of a specific condition or disorder on an individual’s work productivity and daily activities [95]. The WPAI includes three sub-scales. The Work Productivity (WPL) sum-scale measures the degree to which an individual’s work productivity is affected by their condition or disorder, including the number of days missed from work and the number of hours lost while at work. The Activity Impairment (AI) sub-scale measures the degree to which an individual’s ability to perform daily activities is affected by their condition or disorder, including activities related to work, household tasks, and leisure activities. The Overall WPAI score is a combination of the scores from the WPL and AI sub-scales. The WPAI scale can be used in a variety of settings, including clinical trials, to evaluate the effectiveness of a treatment or intervention, and in clinical practice to assess the impact of a particular condition or an individual’s work productivity and daily activities. The WPAI can be used for monitoring productivity at work for patients with migraine [96].

## 4 Conclusions

Clinical scales and PROMs play valuable roles in clinical trials and research settings. Clinical scales and PROMs make evaluating headache disorders more efficient and provide more accurate measurements during follow-up appointments. However, there are concerns about the reliability and validity of these scales because cultural factors may influence a patient’s understanding and responses. They are necessary for regulatory bodies to interpret the results of clinical trials, especially in a disease such as migraine, where there are no objective indicators. A systemic review by Haywood et al. revealed that most PROMs used in migraine treatment were not created using the FDA’s recommended process, which includes patient involvement [17]. Out of 10,903 abstracts of migraine trials, only 46 provided evidence for 20 PROMs. Of these, 11 were specifically migraine related regarding the health impact of migraine (n = 5) or headache (n = 6). The strongest evidence for measurement validity and score interpretation was found in the MSQ v2.1, HIT-6, and PPMQ-R. The authors also noted that the evidence for reliability was limited, but it was still considered acceptable for HIT-6.

There are a small number of PROMs included in product labels, due in part to the requirements that these regulatory bodies place on PROMs to determine that they are “Fit for Purpose.” Currently, only three PROMs have been deemed “Fit for Purpose” by the FDA or European Medicines Agency (EMA) and are included on the labels of migraine treatments. The first is the “Role Function-Restrictive” domain subscale of the MSQ version 2.1, which is included in the label for galcanezumab and atogepant [[97], [98], [99], [100], [101]]. The second is the MPFID domains of “Impact on Everyday Activities” and “Physical Impairment,” which are included in the erenumab label in the USA [102]. The third is the Activity Impairment in Migraine Diary (AIM-D), which is included in the atogepant label [19,103]. In Europe, the EMA has included additional PROMs in the labeling for migraine drugs. HIT-6 and MIDAS are included in the erenumab label, and the Role Function-Restrictive domain of the MSQ v2.1 and MIDAS are included in the galcanezumab label [104,105].

In clinical practice, the IHS uses the 50% response rate as the target criterion for controlled trials of preventive treatment of episodic and chronic migraine in adults [[6], [7], [8]]. In addition, an alternative criterion that is considered acceptable clinically is the demonstration of significant improvement in validated PROMs. Clinically meaningful improvement is evaluated using any of the following validated migraine-specific PROMs. The first is MIDAS, a reduction of ≥5 points when the baseline score is 11–20 or a reduction of ≥30% when the baseline score is > 20. The second is MPFID, a reduction of ≥5 points. The last is HIT-6, a reduction of ≥5 points.

There is an extensive process through which a PROM should be developed and tested. There is a need to develop PROMs to address areas of migraine impact that are commonly reported by patients. Clinical trial results and labeling inclusion of PROMs can be useful to physicians to match patient needs to treatments and set expectations. At this point, physicians have to take into account the impersonalized effect of PROMs and must substitute personal variables. On the other hand, we need more comprehensive studies to test the best method for each expectation. Our latest book published in Springer, **Clinical Scales for Headache Disorders,** will summarize current knowledge and give a reference about the set of scales [106].

## Declaration of competing Interest

The authors declare that they have no known competing financial interests or personal relationships that could have appeared to influence the work reported in this paper.

## Abbreviations

ICHD-3: International classification of headache disorders, 3rd edition
GBD: Global Burden of Disease
PROMs: Patient Reported Outcome Measures
HRQoL: Health-related quality of life
WPAI: Work Productivity and Activity Impairment
FDA: Food and Drug Administration
MCIC: Minimal clinically important change
MID: Minimal important difference
ED: Emergency Department
BHS: The Brief Headache Screen
ID Migraine: Identify Migraine
MS-Q: Migraine Screen Questionnaire
ID-CM: Identify Chronic Migraine
MOH: Medication overuse headache
EM: Episodic migraine
HIT-6: Headache Impact Test-6
SF-36: Short form-36
MIDAS: Migraine Disability Assessment Test
PedMIDAS: A pediatric version of MIDAS
FIS: Functional Impairment Scale
MPFID: Migraine Physical Function Impact Diary
MFIQ: Migraine Functional Impact Questionnaire
PF: Physical Function
UA: Usual Activities
SF: Social Function (SF)
EF: Emotional Function
EQ-5D: EuroQoL Quality of Life Scale
MSQ v2.1: Migraine Specific Quality of Life version 2.1
VAS: Visual analog scale
RR: Role-function-restrictive
PR: Role-function-preventive
MPQ-5: Migraine Prevention Questionnaire
MTSM: Migraine Treatment Satisfaction Measure
PPMQ-R: Migraine Questionnaire-Revised
CORS: Completeness of Response to migraine therapy
PHQ-4: Patient Health Questionnaire-4
PHQ-9: Patient Health Questionnaire-9
BDI: Beck Depression Inventory-II
HRSD: Hamilton Psychiatric Rating Scale
MMPI: Minnesota Multiphasic Personality Inventory
HADS: Hospital Anxiety and Depression Scale
STAI: State-Trait Anxiety Inventory
GAD-7: Generalized Anxiety Disorder-7
C-SSRS: Columbia-Suicide Severity Rating Scale
PGIC: Patient Global Impression
AI: Activity Impairment
